# Factors influencing Purdue Pegboard test results among hand-arm vibration-exposed workers

**DOI:** 10.1093/occmed/kqag023

**Published:** 2026-04-16

**Authors:** A Stjernbrandt, I Liljelind, H Pettersson

**Affiliations:** Department of Epidemiology and Global Health, Umeå University, 901 87 Umeå, Sweden; Department of Epidemiology and Global Health, Umeå University, 901 87 Umeå, Sweden; Department of Epidemiology and Global Health, Umeå University, 901 87 Umeå, Sweden

## Abstract

**Background:**

Manual dexterity can be assessed using the Purdue Pegboard test (PPT) and used for staging of hand-arm vibration (HAV) syndrome.

**Aims:**

To describe PPT results among HAV-exposed workers and investigate what factors influence the results.

**Methods:**

We recruited workers from companies and an occupational medicine clinic in Sweden. They responded to a survey, underwent neurosensory testing and completed the PPT.

**Results:**

The study recruited 225 workers with a mean (SD) HAV exposure duration of 16.7 (13.4) years. The mean (SD) result of the PPT was 12.8 (2.1) pins for the dominant hand, 12.7 (1.9) pins for the non-dominant hand and 10.6 (1.9) pins for both hands. Older age was consistently associated with poorer results (*P *< 0.001). Male sex was associated with lower scores for the dominant hand (*P *= 0.020) but not the non-dominant hand (*P *= 0.269) or both hands (*P *= 0.218). Current smoking was associated with poorer results for the dominant hand (*P *= 0.003) and both hands (*P *= 0.025), but not the non-dominant hand (*P *= 0.803). Self-reported reduced manual dexterity was associated with lower scores for the dominant hand (*P *< 0.001) and both hands (*P *< 0.001) but not the non-dominant hand (*P *= 0.070). Reduced two-point discrimination was consistently associated with poorer PPT results (*P *< 0.001). Low grip strength was associated with lower scores for the non-dominant hand (*P *= 0.008) and both hands (*P *= 0.025), but not the dominant hand (*P *= 0.310).

**Conclusions:**

Researchers noted lower scores on the PPT among older workers, men and smokers. Self-reported reduced manual dexterity, reduced two-point discrimination and low grip strength were also associated with poorer results.

Key learning points

**What is already known:** 

- Careful clinical examination is needed to diagnose hand-arm vibration syndrome.
- The Purdue Pegboard test is useful for assessing manual dexterity and neurosensory staging.
- There is limited knowledge of the factors influencing the outcome of the PPT among HAV-exposed workers.

**What this study adds:** 

- Our study demonstrates that workers exposed to HAV perform worse on the PPT compared to previous studies on other manual workers.
- The results of the PPT are affected by age, sex, smoking, reduced two-point discrimination and low grip strength.
- Self-reported reduced manual dexterity showed insufficient sensitivity using the PPT as the reference method.

**What impact can this study have on practice or policy:** 

- Since our study shows that the PPT results are associated to both subjective and objective measures of manual dexterity, we advocate that the test should be implemented in guidelines for medical surveillance of HAV-exposed workers.

## Introduction

Handheld vibrating tools can damage the peripheral nerves of the hand, and this is commonly called hand-arm vibration syndrome (HAVS) [1]. In Sweden, this is the most frequently compensated occupational disease and a major challenge in the occupational health and safety (OHS) sector [2]. Early recognition is crucial, and medical surveillance is one potential remedy. The Swedish Work Environment Authority regulates medical surveillance, mandating an appointment to an occupational physician every third year for clinical investigation of hand function [3]. However, every other examination can be replaced by a simple screening survey that asks about neurosensory and vascular symptoms. This procedure has been formalised in national guidelines that were developed by the Swedish Trade Association for Occupational Health Services [4]. In a series of papers, we have previously evaluated the diagnostic test performance of the screening survey and the currently used clinical tests [5, 6]. However, no one has systematically investigated the Purdue Pegboard test (PPT) in this setting.

Originally developed in the 1940s, the PPT was intended to evaluate manual dexterity in workers being employed to perform assembly-line work [7]. In the United Kingdom, the PPT has been included for evaluation of dexterity in the standardised Medical Assessment Process of HAVS [8], and the test is also recommended in the new International Consensus Criteria for diagnosing and staging neurosensory HAVS [9]. The main benefit of including the PPT is to obtain a quantitative measure of manual dexterity necessary for staging the condition, both in the older but commonly used Stockholm workshop scale [10] and in the new proposed International Consensus Criteria [9]. However, there are few published studies on the PPT among HAV-exposed workers [11, 12], and there is limited understanding of what other factors influence the outcome of the PPT among manual workers. Furthermore, the agreement between the manual dexterity screening question and the PPT has not been evaluated.

The aim of our study was therefore to describe the outcome of the PPT among workers exposed to HAV, to investigate the factors that influence the results and to evaluate the diagnostic test performance of a survey item for manual dexterity.

## Methods

Our study recruited construction workers, mechanics and welders in northern Sweden who were all using vibrating handheld tools [13]. We also recruited HAV-exposed patients from the Occupational and Environmental Medicine Clinic at the University Hospital of Umeå. All workers responded to a written survey to collect data on age, sex, height, weight, tobacco use and HAV exposure. The survey also included the currently used screening survey for neurosensory HAVS (Table S1, available as Supplementary material at *Occupational Medicine* Online), which included one question about manual dexterity: ‘Do you have any of the following: difficulty with fastening buttons?’. The worker could respond: ‘No’; ‘Insignificant’; ‘Somewhat’; or ‘Quite a lot’. The current routine in clinical practice is to interpret ‘Somewhat’ or ‘Quite a lot’ as a positive response, to be followed up by clinical examination [4]. Clinical testing was performed either by two of the authors (I.L. and H.P.) or by a physician at the clinic [5]. Vibrotactile quantitative sensory testing was performed by one of the authors (H.P.) or by clinic staff [6]. All testing adhered to national guidelines of the Swedish Trade Association for Occupational Health Services [4]. To reduce the inter-rater variability, we used strict examination protocols, limited the number of examiners and conducted examiner training before starting the study. The Swedish Ethical Review Authority (DNR 2021-01463) approved the study protocol.

The testing was performed in a quiet room with normal indoor temperature (20–22 °C), with the worker sitting comfortably at a table at waist height. Hands were heated with warm water if the finger skin temperature was below 28.0 °C at the start of testing. We used the PPT (model 32020, Lafayette Instrument, Lafayette, IN, USA) with two parallel lines of holes (25 each, 1 cm spacing) and fifty metal pins located at the top of the board. The subject was instructed to place as many pegs into the holes as possible during 30 s; first with the dominant hand, then the non-dominant hand and lastly with both hands [14]. The workers had one practice run for each test (dominant, non-dominant and both hands), after which the PPT was conducted once. If the worker dropped a pin, they were instructed not to pick it up. Placing <12 pins for the dominant and non-dominant hands, and <10 pins using both hands was considered abnormal [9]. Static two-point discrimination was established on the volar aspect of the distal phalanx of the index fingers, using a standardised discriminator (Touch-Test, North Coast Medical, Gilroy, CA, USA). Three attempts were made, and two correct responses were required for a specific reading. Grip strength was recorded using a hydraulic hand dynamometer (Jamar, Sammons Preston, Rolyan, Bolingbrook, IL, USA), with the worker seated, the elbow bent ∼90 degrees and the dynamometer handle at the second smallest grip position. The mean value out of three attempts was used. Vibrotactile quantitative sensory testing was conducted using the Vibro-Sense Meter II (VibroSense Dynamics, Malmö, Sweden), performed on the volar aspect of the distal phalanx of the index fingers and the results presented as the deviation from the mean value (Z average) of an age- and sex-matched reference population [15].

The study sample size was determined to achieve precision in estimating sensitivity and specificity, assuming a 40% prevalence of abnormal findings during clinical testing in at least one sensory modality, based on results from previous studies in the Scandinavian setting [16, 17]. Using the Buderer equation [18], we estimated that 202 participants were required to achieve a 10% target margin of error. The results of the PPT were normally distributed and presented as mean values with standard deviation (SD). Categorical data were presented as numbers (*n*) and valid percentages. Missing data were excluded from analyses. Spearman’s rank correlation coefficient (*r*_s_) was used to test the correlation between covariates. General linear models with fixed intercepts were used, with independent variables selected a priori based on the authors’ understanding of factors that could influence the PPT outcome. Sensitivity, specificity, positive and negative predictive values, positive and negative likelihood ratios and diagnostic odds ratios were calculated using the PPT results as the reference method. The predictive values were calculated only for company-recruited workers, as they are strongly dependent on the prevalence of the condition, which we anticipated would be higher among clinic-recruited workers.

## Results

The study recruited 225 workers, of whom 17 (8%) were women. The study population consisted of 150 (67%) company-recruited and 75 clinic-recruited workers. The participation rate of company-recruited workers could not be calculated due to the convenience sampling approach; it was 41% for clinic-recruited workers. Data on pre-existing diseases have previously been published [6], but in brief, 21 subjects (9%) reported previous neurological conditions such as carpal tunnel syndrome. The mean (SD) age was 40.6 (13.7) years, and the mean (SD) exposure duration to HAV was 16.7 (13.4) years. There was a strong correlation between age and exposure duration (*r*_s_=0.81; *P* < 0.001). The mean (SD) body mass index (BMI) was 27.3 (4.5). Ten (5%) were current smokers, 62 (28%) were previous smokers and 152 (68%) were never smokers. For the survey item asking about difficulties fastening buttons, 127 (56%) answered ‘No’, 38 (17%) ‘Insignificant’, 47 (21%) ‘Somewhat’ and 13 (6%) ‘Quite a lot’. The PPT was completed by 200 (89%) workers, with results presented in Table 1. In brief, the mean (SD) result for all workers was 12.8 (2.1) pins for the dominant hand, 12.7 (1.9) pins for the non-dominant hand and 10.6 (1.9) pins for both hands.

**Table 1:** Results of Purdue Pegboard testing categorized by age, sex and survey responses

		Dominant hand	Non-dominant hand	Both hands	Sum of dominant, non-dominant and both hands
	*n* (%)	Mean (SD)	Mean (SD)	Mean (SD)	Mean (SD)
All	225 (100.0)	12.8 (2.1)	12.7 (1.9)	10.6 (1.9)	36.1 (5.2)
Age group (years)					
19–35	98 (44)	13.2 (2.1)	13.0 (1.8)	11.0 (1.7)	37.3 (4.6)
36–51	58 (26)	13.2 (1.8)	13.3 (1.5)	11.1 (1.8)	37.6 (4.3)
52–67	69 (31)	11.8 (2.1)	11.5 (2.0)	9.6 (1.9)	32.9 (5.5)
Sex					
Male	208 (92)	12.7 (2.1)	12.6 (1.9)	10.6 (1.9)	35.9 (5.1)
Female	17 (8)	14.2 (2.4)	13.3 (1.8)	11.6 (1.9)	39.1 (5.6)
Difficulty fastening buttons					
No	127 (56)	13.5 (1.9)	13.1 (1.8)	11.1 (1.7)	37.7 (4.6)
Insignificant	38 (17)	12.2 (1.6)	12.4 (1.5)	10.6 (1.5)	35.2 (3.8)
Somewhat	47 (21)	11.9 (2.0)	11.7 (2.1)	9.7 (1.9)	33.4 (5.4)
Quite a lot	13 (6)	9.9 (2.3)	11.5 (2.2)	8.2 (1.8)	29.6 (5.3)

We then constructed multiple linear regression models for personal factors (Table 2) and clinical characteristics (Table 3) with the PPT results as the dependent variable. Univariate linear regression results are presented in Tables S2 and S3 (available as Supplementary material at *Occupational Medicine* Online). HAV exposure duration could not be included in the model due to strong covariance with age. Among personal factors, older age was consistently associated with poorer results (*P *< 0.001 for all). Male sex was associated with lower scores for the dominant hand (*P *= 0.020). Current smoking was associated with poorer results for the dominant hand (*P *= 0.003) and both hands (*P *= 0.025). BMI and having diabetes were not associated with the PPT results. Regarding clinical characteristics, the highest response alternative (‘Quite a lot’) for the survey question about reduced manual dexterity was associated with a poorer PPT result for the dominant hand (*P* < 0.001) and both hands (*P* < 0.001). Reduced two-point discrimination in the index finger was consistently associated with poorer PPT results (*P *< 0.001 for all). Low grip strength was associated with lower scores for the non-dominant hand (*P *= 0.008) and both hands (*P *= 0.025). Reduced vibration detection thresholds were not associated with the PPT results.

**Table 2:** Multiple linear model for personal factors in relation to results on the Purdue Pegboard test

		Dominant hand			Non-dominant hand			Both hands		
Predictor		*β*	SE	*P* value	*β*	SE	*P* value	*β*	SE	*P* value
Age (years)	19–67 (continuous)	−0.04	0.01	<0.001	−0.04	0.01	<0.001	−0.04	0.01	<0.001
Sex	Female (*n* = 17)	Ref	–	–	Ref	–	–	Ref	–	–
	Male (*n* = 208)	−1.34	0.57	0.020	−0.58	0.53	0.269	−0.63	0.51	0.218
Body mass index (kg/m²)	19.8–47.3 (continuous)	0.01	0.03	0.787	−0.01	0.03	0.654	−0.03	0.03	0.257
Smoking	Never (*n* = 152)	Ref	–	–	Ref	–	–	Ref	–	–
	Former (*n* = 62)	−0.23	0.33	0.485	−0.62	0.30	0.042	−0.01	0.29	0.979
	Current (*n* = 10)	−2.08	0.69	0.003	−0.16	0.64	0.803	−1.38	0.61	0.025
Diabetes	No (210)	Ref	–	–	Ref	–	–	Ref	–	–
	Yes (*n* = 15)	−0.42	0.59	0.485	−0.36	0.55	0.514	−0.72	0.53	0.175
*R* ^2^		0.16			0.13			0.16		

Abbreviations: *β*: regression coefficient, *R*^2^: adjusted explained variation, SE: standard error.

**Table 3:** Multiple linear model for clinical characteristics in relation to results on the Purdue Pegboard test

		Dominant hand			Non-dominant hand			Both hands		
Predictor		*β*	SE	*P* value	*β*	SE	*P* value	*β*	SE	*P* value
Difficulty fastening buttons	Not at all (*n* = 127)	Ref	–	–	Ref	–	–	–	–	–
	Insignificant (*n* = 38)	−0.94	0.40	0.021	−0.50	0.35	0.156	−0.13	0.36	0.716
	Somewhat (*n* = 47)	−1.02	0.40	0.011	−0.81	0.36	0.024	−0.83	0.35	0.020
	Quite a lot (*n* = 13)	−2.83	0.68	<0.001	−1.11	0.61	0.070	−2.34	0.60	<0.001
Two-point discrimination (mm)	2–8 (continuous)	−0.55^a^	0.16	<0.001	−0.55^b^	0.15	<0.001	−0.48^a^	0.14	<0.001
Grip strength (kg)	11–80 (continuous)	0.01^a^	0.01	0.310	0.03^b^	0.01	0.008	0.02^a^	0.01	0.025
Vibration detection threshold (Z average)	−2.5–6.6 (continuous)	0.04^a^	0.16	0.802	0.10^b^	0.13	0.443	0.05^a^	0.14	0.742
*R* ^2^		0.24			0.21			0.23		

a Tested on the dominant hand.

b Tested on the non-dominant hand.

Abbreviations: *β*: regression coefficient, *R*^2^: adjusted explained variation, SE: standard error.

The diagnostic test performance of the survey question for manual dexterity, using the PPT as the reference method, is presented in Table S4. When interpreting ‘Somewhat’ or ‘Quite a lot’ as a positive response, as is the current routine among Swedish OHS providers, the sensitivity ranged from 40 to 44% and the specificity from 83 to 85%, depending on which hand was tested. Using only ‘Quite a lot’ as a positive response lowered sensitivity and increased specificity.

## Discussion

When performing the PPT, we found a mean (SD) of 12.8 (2.1) pins for the dominant hand, 12.7 (1.9) pins for the non-dominant hand and 10.6 (1.9) pins for both hands. To compare, Gerhardsson et al. performed a modified PPT on HAV-exposed workers (*n* = 47) and reported median values of 13 pins for the dominant hand and 12 pins for the non-dominant hand, which were both significantly poorer than the results of unexposed referents (*n* = 18) [11]. Bovenzi *et al.* [12] performed the PPT on HAV-exposed workers (*n* = 249) and reported median (IQR) values of 13 (2) pins for the dominant hand, 13 (2) pins for the non-dominant hand and 10 (2) pins for both hands, thus closely resembling our results. Based on the same original data collection, Poole *et al.* [9] reported on normal values for heavy manual workers (mostly maintenance operators) that were not HAV-exposed (*n* = 118) and found a mean (SD) of 14.9 (1.6) pins for the dominant hand, 14.6 (1.5) pins for the non-dominant hand and 12.8 (1.5) pins for both hands. Basing the limit of normality on 2 SD below the mean, an appropriate cut-off would be 12 pins for both the dominant and non-dominant hands and 10 pins for both hands: this was used in our analyses of diagnostic test performance. Overall, the test performance was reduced in our study compared to that of Poole *et al.*, ∼2 pins less per round of tests. In another normative study, Stijic *et al.* [19] reported PPT results from randomly selected men and women (*N* = 1355) and for men between 40 and 49 years, which was most comparable to our study, the mean (SD) was 14.2 (1.6) pins for the right hand (where 92% were dominant), 13.8 (2.2) pins for the left hand and 11.7 (2.7) pins for both hands. This was very similar to the results of Poole *et al.* [9] and, once again, reflected better performance than we found. Thus, when compared to reference studies, both our results and those of Bovenzi *et al.* [12] suggest that HAV-exposed workers perform worse on the PPT than unexposed individuals, including those with other types of heavy manual work. This may be due to the detrimental mechanical effects of HAV exposure on the neurosensory and motor function of the hands, but could also have other explanations, such as differences in sampling for the studies.

We explored other factors that could influence the result of the PPT and found that older age, male sex and current smoking were associated with poorer results, while BMI and diabetes were not statistically significant covariates. Self-reported reduced manual dexterity, reduced two-point discrimination, and low grip strength were generally associated with worse PPT results. Our findings corroborate those of previous studies showing an effect of age and sex [20–22]. This is intuitive, since ageing affects both the neurosensory, musculoskeletal and visual performance, while the sex difference can be explained by differences in finger size [23]. Performing the PPT does not require a lot of manual force per se, but grip strength is likely a proxy marker for global motor function in the hand. We found that performance was generally better with the dominant hand, which is also consistent with previous research [20, 21, 24]. Stijic *et al.* [19] explored covariates for the PPT and found that diabetes was consistently associated with poorer results. In their study, BMI only affected the testing of the left hand and both hands, but not the right hand. In comparison, we found no effect of diabetes or BMI in either hand.

We also evaluated the diagnostic test performance of the screening question, using the PPT as the reference method with the cut-offs determined by Poole *et al.* [9]. We used both the current interpretation of the screening question (where ‘Somewhat’ or ‘Quite a lot’ is considered a positive response) as well as a stricter interpretation (where only ‘Quite a lot’ is considered positive). Regardless, the sensitivity was so low for the screening question (12–44%) that it would lead to many false-negatives, i.e. having workers with reduced manual dexterity based on the PPT classified as normal based on the survey response. However, the specificity of the screening question was acceptable (83–99%). A high prevalence of abnormal PPT results tended to increase the positive and decrease the negative predictive value. If the survey was to be used for screening on younger workers, a lower proportion of abnormal findings would be expected, leading to poorer predictive values. Positive likelihood ratios above 10 strongly rule in disease [25] but this was not found when using ‘Somewhat’ or ‘Quite a lot’ as positive response alternatives, where values ranged from 2.4 to 2.9. Neither did the negative likelihood ratios provide strong evidence for ruling out disease. However, the positive likelihood ratio and diagnostic odds ratio were sufficiently improved by only using the highest response alternative (‘Quite a lot’) as a positive response, but this led to an unacceptable drop in sensitivity. Across all indices of diagnostic performance, the screening question did not perform well enough to substitute for clinical testing, as is the current routine in Sweden. In future iterations, this question should be modified or complemented with other questions about manual dexterity, and the response alternatives should also be revised, as they are currently perceived as skewed towards negative options [5].

We used convenience sampling for recruitment and do not know whether symptomatic workers preferentially chose to participate. On the other hand, workers with HAVS or other occupational injuries may already have left the workplace, leading to an opposite selection bias (i.e. the healthy worker effect). We also recruited HAV-exposed workers from the clinic, and these subjects had slightly more abnormal test results (data not shown). We decided to recruit from both companies and the clinic to get a representative sample of what an occupational physician might encounter in their practice, and we believe that our results are generalisable to the OHS setting. Despite our best efforts, we were only able to recruit very few women, so any analyses with regard to sex should be interpreted cautiously. The multiple linear models (Tables 2 and 3) showed low explained variation (*R*^2^), which indicates that there are other factors that affect the PPT that were not considered in our study.

Manual dexterity has previously been defined as the ability to make rapid, skilful, controlled manipulative movements of small objects, where the fingers are primarily involved [26]. However, the concept of manual dexterity is complex, and a poor performance can reflect both reduced neurosensory function, motor deficits, poor eyesight or limited attention to the task at hand. While most clinical tests used to examine HAV-exposed workers assess specific sensory modalities, the PPT assesses a more global function [27]. All clinical tests should be acknowledged as subjective and considered in the context of the overall clinical presentation rather than definitive indicators of abnormality or normality [27]. According to the manufacturer of the PPT, age, gender and hand preference need to be considered when evaluating test scores. In future studies, we suggest that occupational factors (and particularly HAV exposure) should also be considered, to gain a better understanding of how physical exposures affect dexterity. One important limitation is the fact that the workers in our study performed the PPT only once. Previous research suggests a practice effect, in which subjects improve with repeated trials [21], especially among young individuals [28]. Also, the supervision of the PPT is crucial, to understand what is limiting functional capacity during the test (e.g. eyesight or tremor). Unfortunately, we did not collect data on such factors. Strengths of our study include a rather large sample size, standardised assessment using detailed protocols that followed national guidelines [4] and a limited number of examiners to reduce inter-rater variability.

We believe that performing the PPT is helpful when diagnosing HAVS, especially for neurosensory staging, which can affect recommendations for future work and workers’ compensation claims. It is also important for detecting reduced manual dexterity, for which there is no other clinical test in the current national guidelines and where the screening question performs poorly. We therefore advocate that the PPT should be included in the national guidelines for medical surveillance of HAV-exposed workers and performed routinely. The PPT equipment is not expensive, but time constraints may limit its implementation in OHS practices. It also requires a trained medical professional for instruction and supervision. However, these challenges could easily be overcome if the test was mandated in the national guidelines.

We conclude that lower PPT scores were found among older workers, men and smokers. Self-reported reduced manual dexterity, reduced two-point discrimination and low grip strength were also associated with poorer results. The sensitivity of the screening question for manual dexterity in relation to the PPT was low, while the specificity was high.

## Supplementary Material

kqag023_Supplementary_Data

## Data Availability

The dataset used in our study can be made available upon reasonable request to the corresponding author.
