# Supplementary material for: Factors influencing Purdue Pegboard test results among hand-arm vibration-exposed workers
Source: Occup Med (Lond). 2026 Apr 16;76(3):203–9. doi: 10.1093/occmed/kqag023 (PMC13261060; doi:10.1093/occmed/kqag023)
Supplement: kqag023_Supplementary_Data [file kqag023_supplementary_data.zip › Supplementary_Table_S3.pdf]

Supplementary Table S3. Simple linear regression of clinical characteristics in relation to results on the Purdue pegboard test

		Dominant hand				Non-dominant hand				Both hands			
**Predictor**		**β**	**SE**	***p* value**	**R^2**	**β**	**SE**	***p* value**	**R^2**	**β**	**SE**	***p* value**	**R^2**
Difficulty fastening buttons	Not at all (n=127)	Ref	-	-	0.20	Ref	-	-	0.10	Ref	-	-	0.17
	Insignificant (n=38)	-1.23	0.37	0.001		-0.78	0.36	0.031		-0.48	0.34	0.159	
	Somewhat (n=47)	-1.55	0.36	<0.001		-1.42	0.35	<0.001		-1.39	0.33	<0.001	
	Quite a lot (n=13)	-3.57	0.62	<0.001		-1.64	0.60	0.007		-2.92	0.57	<0.001	
Two-point discrimination (mm)	2–8 (continuous)	-0.80[a]	0.15	<0.001	0.13	-0.74[b]	0.14	<0.001	0.13	-0.68[a]	0.13	<0.001	0.12
Grip strength (kg)	11–80 (continuous)	0.02[a]	0.01	0.095	0.01	0.04[b]	0.01	<0.001	0.07	0.03[a]	0.01	0.002	0.04
Vibration detection threshold (Z average)	-2.5–6.6 (continuous)	-0.40[a]	0.14	0.004	0.04	-0.15[b]	0.13	0.228	<0.01	-0.24[a]	0.13	0.061	0.01

[a] Tested on the dominant hand. [b] Tested on the non-dominant hand.

β: regression coefficient, SE: standard error, R^2: explained variation.
