# Supplementary material for: Factors influencing Purdue Pegboard test results among hand-arm vibration-exposed workers
Source: Occup Med (Lond). 2026 Apr 16;76(3):203–9. doi: 10.1093/occmed/kqag023 (PMC13261060; doi:10.1093/occmed/kqag023)
Supplement: kqag023_Supplementary_Data [file kqag023_supplementary_data.zip › Supplementary_Table_S1.pdf]

Supplementary Table S1. The Swedish national screening survey for neurosensory symptoms among workers exposed to hand-arm vibration

Do you have any of the following? (Refers to when you are not working with vibrating tools)

	No	Insignificant	Somewhat	Quite a lot
Impaired ability to feel touch in fingers/hand?	☐	☐	☐	☐
Impaired ability to feel heat in fingers/hand?	☐	☐	☐	☐
Impaired ability to feel cold in fingers/hand?	☐	☐	☐	☐
Impaired ability to feel vibration in fingers/hand?	☐	☐	☐	☐
Reduced strength in fingers/hand?	☐	☐	☐	☐
Numbness/tingling in fingers/hand?	☐	☐	☐	☐
Pain when cold in fingers/hand?	☐	☐	☐	☐
Difficulty with fastening buttons?	☐	☐	☐	☐
Clumsiness?	☐	☐	☐	☐
Pain/aches in fingers/hands/lower arm/elbow?	☐	☐	☐	☐
Pain/aches in neck/shoulder?	☐	☐	☐	☐

The survey has been translated from Swedish to English.
