# Supplementary material for: Factors influencing Purdue Pegboard test results among hand-arm vibration-exposed workers
Source: Occup Med (Lond). 2026 Apr 16;76(3):203–9. doi: 10.1093/occmed/kqag023 (PMC13261060; doi:10.1093/occmed/kqag023)
Supplement: kqag023_Supplementary_Data [file kqag023_supplementary_data.zip › Supplementary_Table_S2.pdf]

Supplementary Table S2. Simple linear regression of personal factors in relation to results on the Purdue pegboard test

		Dominant hand				Non-dominant hand				Both hands			
**Predictor**		**β**	**SE**	***p* value**	**R^2**	**β**	**SE**	***p* value**	**R^2**	**β**	**SE**	***p* value**	**R^2**
Age (years)	19–67 (continuous)	-0.05	0.01	<0.001	0.08	-0.04	0.01	<0.001	0.10	-0.05	0.01	<0.001	0.11
Sex	Female (n=17)	Ref	-	-	0.03	Ref	-	-	0.01	Ref	-	-	0.02
	Male (n=208)	-1.51	0.57	0.009		-0.66	0.53	0.216		-1.00	0.52	0.054	
Body mass index (kg/m²)	19.8–47.3 (continuous)	-0.06	0.03	0.079	0.01	-0.05	0.03	0.068	0.01	-0.08	0.03	0.004	0.04
Smoking	Never (n=152)	Ref	-	-	0.05	Ref	-	-	0.02	Ref	-	-	0.03
	Former (n=62)	-0.19	0.34	0.577		-0.65	0.31	0.038		-0.05	0.30	0.859	
	Current (n=10)	-2.18	0.18	0.002		-0.30	0.65	0.646		-1.62	0.64	0.012	
Diabetes	No (210)	Ref	-	-	0.01	Ref	-	-	0.01	Ref	-	-	0.02
	Yes (n=15)	-0.86	0.62	0.169		-0.81	0.57	0.157		-1.12	0.56	0.044	

β: regression coefficient, SE: standard error, R^2: explained variation.
